# Correlations Between Metabolic/Vascular Risk Factors and Plasma Biomarkers to Predict AD Brain Pathology Amongst African Americans

**DOI:** 10.1002/alz70856_102377

**Published:** 2025-12-24

**Authors:** Rejina Roufegarinejad, Joseph Cummings, Charisse N. Winston

**Affiliations:** ^1^ University of San Diego, San Diego, CA, USA; ^2^ USC ATRI, San Diego, CA, USA; ^3^ University of Southern California, Alzheimer's Disease Therapeutic Research (ATRI), San Diego, CA, USA

## Abstract

**Background:**

The impact of metabolic and vascular factors such as heart disease, hypertension, type 2 diabetes, and obesity on the risk of Alzheimer's disease (AD) is becoming increasingly acknowledged, particularly among metabolically unhealthy, overweight/obese (MUO) individuals. However, an increased risk of AD among metabolically healthy, overweight/obese (MHO) individuals is not well understood. Body Mass Index (BMI) is a commonly used metric for assessing the risk of adverse health outcomes; however, current standards do not correlate equally across racial and ethnic groups, especially among Black or African Americans.

**Method:**

Multiple linear regression models at baseline were employed to determine BMI/metabolic profiles of MUO and MHO Black individuals from the Alzheimer's Disease Neuroimaging Initiative (ADNI) correlated with biomarkers of neurodegeneration and AD risk.

**Result:**

Since 2004, ADNI has enrolled 488 Black participants, of which only 307 Black participants were included (healthy controls: females, n = 188; males, n = 74; mild/moderate AD: female, n = 21; male, n = 24). At baseline, no significant differences between BMI and cognitive status were observed amongst all Black individuals. Preliminarily, a positive yet nonsignificant correlation between BMI and cognitive status, suggesting reduced AD risk, was observed in MHO black men as compared to MHO Black women. Using multivariable linear regression models, cross‐sectional associations of MUO and MHO profiles of Black individuals with plasma biomarkers, brain Aβ load, and hippocampal volume will be further explored and presented.

**Conclusion:**

AD risk may be reduced amongst MHO black men however deeper investigations into the longitudinal changes in cognitive status, Aβ and tau brain pathology, and plasma biomarkers amongst MHO individuals are required.

